# Detection and molecular characterisation of thyroid cancer precursor lesions in a specific subset of Hashimoto's thyroiditis

**DOI:** 10.1038/sj.bjc.6602097

**Published:** 2004-08-03

**Authors:** A Gasbarri, S Sciacchitano, A Marasco, M Papotti, A Di Napoli, A Marzullo, P Yushkov, L Ruco, A Bartolazzi

**Affiliations:** 1 Department of Pathology, St' Andrea Hospital, University La Sapienza, via di Grottarossa 1035, 00189 Rome, Italy; 2 Department of Endocrinology, University La Sapienza and St Peter Hospital Research Center, Associazione Fatebenefratelli for Research, via Cassia 600, 00189 Rome, Italy; 3 Department of Biomedical Sciences and Human Oncology, University of Turin and St Luigi Hospital, Regione Gonzole 10, 10043 Orbassano, Turin, Italy; 4 Department of Pathomorphology, Head Research Center for Endocrinology, Dmitry Ulyanov str. 11, 117036 Moscow, Russia; 5 Department of Oncology-Pathology, Cellular and Molecular Tumor Pathology, CCK R8:04, S-17176, Karolinska Hospital, Stockholm, Sweden

**Keywords:** galectin-3, thyroid cancer, Hashimoto's thyroiditis, laser capture microdissection

## Abstract

Hashimoto's thyroiditis (HT) represents the most common cause of hypothyroidism and nonendemic goiter, but its clinical and pathological heterogeneity opens the question if this disease should be more properly considered as a spectrum of different thyroid conditions rather than as a single nosological entity. In this study, we analysed 133 cases of HT for the expression of galectin-3, a lectin molecule involved in malignant transformation, apoptosis and cell cycle control. An unexpected expression of galectin-3 was demonstrated in a subset of HT together with the presence of HBME-1, c-met and cyclin-D1 that are also involved in malignant transformation and deregulated cell growth. Furthermore, a loss of allelic heterozygosity in a specific cancer-related chromosomal region was demonstrated in some HT harbouring galectin-3-positive follicular cells, by using laser capture microdissection. On the basis of the morphological and molecular findings we identified four subsets of HT: (a) HT with classic features of chronic autoimmune thyroiditis; (b) HT associated to hyperplastic/adenomatous lesions; (c) HT harbouring thyroid cancer precursors; (d) HT associated to unequivocal thyroid microcarcinomas. Our findings provide a well-substantiated morphological and molecular demonstration that HT may include a spectrum of different thyroid conditions ranging from chronic autoimmune thyroiditis to thyroiditis triggered by specific immune-response to cancer-related antigens.

Hashimoto's thyroiditis (HT) represents the most common cause of hypothyroidism and nonendemic goiter. This pathological entity was first described by Hashimoto in 1912 who reported four patients with goiter in whom the histology of the thyroid gland was characterised by diffuse lymphocyte infiltration, atrophy of follicular cells, presence of eosinophilic granular thyrocytes (also worded oncocytic cells or Hurtle's cells) and fibrosis (Hashimoto, 1912). Over 40 years later, presence of autoantibodies was discovered in patients with this disorder and HT was finally categorised as a chronic autoimmune thyroiditis (Roitt *et al*, 1956).

The hallmark of autoimmune thyroiditis is the presence of thyroid-specific autoantibodies in the serum, in particular anti-thyroglobulin antibodies (TG), anti-thyroperoxidase (TPO) antibodies and less frequently anti-TSH receptor antibodies, together with a variable degree of thyroid dysfunction.

Patients affected by HT are generally referred for hypothyroidism or for the presence of a nodular goiter characterised by its firmness and irregular surface. Fine-needle aspiration biopsy (FNA) reveals presence of lymphocytes and plasma cells infiltration, intermingled with eosinophilic granular thyrocytes.

These features, when coexisting with the presence of anti-TPO antibodies, are considered diagnostic in most of the cases. However, it is well known that patients with HT show a rather wide spectrum of clinical and laboratory presentation. The presence of significant serum levels of thyroid-specific autoantibodies, in fact, is not the rule. Furthermore, occurrence of glandular fibrosis and destruction of follicular epithelial cells are of variable intensity and evolution (Dayan and Daniels, 1996). It is noteworthy that only some Hashimoto's goiters become atrophic over the years. Thyroid fibrosis with dense infiltrate of plasma cells represents the final evolution of these cases, but in the majority of HTs, little histological progression has been observed in patients undergoing a second biopsy up to 20 years after the first (Vickery and Hamblin, 1961; Hayashi *et al*, 1985).

Here we present a morphological and molecular analysis of 133 histologically and clinically proven HTs showing a variable degree of inflammation, oncocytic changes, fibrosis and glandular atrophy, which are considered the histological hallmarks of this disease.

Molecular and immunohistochemical studies directed to detect specific gene profiles and related proteins in thyroid cancer have been extensively reported in literature (Bartolazzi *et al*, 2001; Huang *et al*, 2001; Ruco *et al*, 2001; Wreesmann *et al*, 2002). Experimental data, mostly arising from the molecular analysis of papillary thyroid carcinoma (PTC), contributed to identify a panel of genes overexpressed in thyroid malignancy. Among these genes, FN-1 (fibronectin-1), KRT-19 (keratin-19) and MET (hepatocyte growth factor receptor) gene have been found consistently overexpressed in PTC (Huang *et al*, 2001).

Overexpression of c-met protein has been reported as a distinguishing feature of almost every case of well-differentiated PTC, but interestingly a moderate expression of this molecule was observed in peritumoral non-neoplastic follicles as well as in some HTs (Ruco *et al*, 2001). Research in this field has recently been focused on LGALS3 gene that codes for the *β*-galactosil binding protein called galectin-3. This lectin molecule, which is expressed in the vast majority of PTC, as well as in follicular carcinoma, is directly involved in transformation of follicular thyroid cells and in regulation of apoptosis (Bartolazzi *et al*, 2001; Huang *et al*, 2001; Takenaka *et al*, 2003; Yang and Liu, 2003).

For its pattern of expression in thyroid cancer, galectin-3 has recently been proposed as diagnostic marker of malignant transformed thyrocytes (Bartolazzi *et al*, 2001).

In a subset of HTs assessed here, superimposed alterations of follicular thyroid cells, ranging from focal cytoarchitectural atypia to microcarcinoma were morphologically identified. Interestingly, the vast majority of these lesions expressed galectin-3.

These findings raise the question if some HTs may be triggered by specific immunoresponse to transformed thyrocytes.

Detection of both galectin-3-positive atypical areas and microcarcinomas, in a subset of HTs, supports the hypothesis that precursor lesions of thyroid cancer may be present in these thyroid conditions. To date, the diagnosis of HT has been mostly based on clinical, immunological and histomorphological criteria, but it seems that this conventional diagnostic approach only shows ‘one side of the moon’.

## MATERIALS AND METHODS

### Histological samples and HT subclassification

A total of 133 cases of clinically and pathologically defined HTs, collected from different Institutions, were tentatively subclassified by using the following morphological criteria: (i) HTs with classical features of chronic autoimmune thyroiditis: in this group fall those cases of HT, in which a diffuse lymphocyte infiltrate, sometimes organized in nodules or follicles, was associated to oncocytic changes and to a variable degree of fibrosis and atrophy of thyroid parenchyma; (ii) HTs with coexistence of benign proliferating thyroid lesions: this cluster included HTs associated to hyperplastic nodules or adenomas; (iii) HTs with focal atypical or suspicious areas: this cluster includes those HTs in which microscopic lesions of dubious interpretation were detected. These suspicious areas consisted of thyrocytes arranged in follicles or in solid cell nests, showing the nuclear features of PTC (nuclear clearing and groves) but without any evidence of malignancy. Such suspicious thyroid cells were mostly intimately associated with reactive lymphoid nodules, or were placed in the context of sclerotic areas; (iv) HTs with unequivocal microscopic foci of thyroid cancer: when focal or occult thyroid malignancies were discovered in HTs. Most of the detected microcarcinomas showed features of PTC with size less then 4 mm in diameter. In isolated cases, follicular variants of PTC were detected. Both the original diagnosis and subclassification of HTs were confirmed by at least two independent pathologists.

### Monoclonal antibodies and immunochemical assay

Formalin-fixed and paraffin-embedded tissue specimens were used to prepare seriate tissue sections for conventional morphologic analysis as well as for phenotypic and molecular studies. Purified rat monoclonal antibody (mAb) to galectin-3 (Mabtech, Nacka, Sweden), mouse mAbs to cyclin D1 (Novocastra Laboratories Ltd, Newcastle upon Tyne, UK), mAbs to HBME-1 and to c-met protein (Dako Corporation, Carpinteria, CA, USA) were used in immunohistochemistry according to the manufacturer's instructions.

Antigen-retrieval microwave treatment of tissues slides in 0.01 mol l^−1^ citrate buffer pH 6,0 was applied as required. To minimise the occurrence of false positive results, a biotin-free immunoperoxidase staining method was considered in this study. This was obtained by using horseradish-peroxidase conjugated (HRP-conjugated) rabbit anti-mouse and rabbit anti-rat immunoglobulins as secondary antibodies, in indirect immunoperoxidase assay (Dako).

Briefly, slides were incubated overnight with selected monoclonal antibodies at 4°C in a moist chamber. Purified rat mAb directed to galectin-3 was used at a concentration range of 5–10 *μ*g ml^−1^. Mouse mAbs to c-met protein, HBME-1 and cyclin-D1 were used diluted 1 in 20 to 1 in 50, depending on the antibody concentration in the batch. After incubation with the appropriate HRP-conjugated secondary antiserum, the enzymatic activity was visualised with 3,3′-diamino-benzidine (Dako). Finally, slides were counterstained with Mayer's haematoxylin and mounted in Eukitt (Bioptica, Milan, Italy) for microscopy.

Positive cases were classified as +/− when a weak and heterogeneous immunostaining was restricted to isolated follicular cells or cell clusters; and + when a homogeneous staining of all the lesion was visible. Immunohistochemical evaluations were carried out independently by two experienced pathologists.

### Laser capture microdissection and loss of heterozygosity (LOH) analysis

Loss of heterozygosity analysis at 7q32–34 was carried out focusing on the cluster of HTs with morphologically suspicious galectin-3-positive areas.

Seriate tissue sections were obtained from a total of 10 HTs and were subjected to laser microdissection and molecular analysis with the aim to detect specific genetic alterations. Two cases of classical HTs (galectin-3 negative) were used as control. In these cases, apparently normal follicles surrounded by a diffuse lymphocyte infiltrate were microdissected. In all the other instances, galectin-3-positive focal undefined lesions or unequivocal thyroid microcarcinomas were specifically microdissected. Selection and isolation of the candidate cells and/or microscopic areas to be captured was performed under direct microscopic visualisation, by using the Pix Cell II Laser Capture Microscope (Arcturus Engineering, Mountain View, CA, USA). Approximately 500 cells were captured from each lesion and collected onto a dedicated cap. All caps were handled with gloves to reduce contamination and kept separately from the others during all the following experimental procedures. After microdissection and DNA extraction, polymerase chain reaction (PCR) was carried out in the presence of specific primers flanking the sequence of two microsatellite markers (D7S-1779 and D7S-2468) located at the chromosomal region 7q32–34 (cytogenetic localization 150–152 cM) previously reported to be altered in thyroid cancer (Zhang *et al*, 1998; Roque *et al*, 2001).

Amplification of the microsatellite markers was obtained with the following primers: D7S-1779-Forward 5′-ACCGTGAATACGCCAAACT-3′, D7S-1779-Reverse 5′-AGAAGATTCAAAAGTGAAGAGTTACA-3′; D7S-2468-Forward 5′-TTTCACAAGAGGCCACCTTC-3′, and D7S-2468-Reverse 5′-CGCACCTTACCAATGTGACTT-3′.

Polymerase chain reaction amplifications of DNA were performed by means of the hot start technique in a 20 *μ*l reaction mixture containing 200 *μ*M dNTP mix, 0.6 *μ*M primers, and PCR reaction buffer (containing 1.25 mM MgCl_2_). Polymerase chain reaction amplifications were run in the Thermal Cycler PCR GeneAmp System 2400 (Perkin Elmer Corp., Norwalk, CT, USA). The reactions consisted in an initial denaturation step at 94°C for 5 min at the end of which 1 U of *Taq* polymerase (Perkin–Elmer Corp., Norwalk, CT, USA) was added, followed by 35–40 cycles. Each cycle was composed of a denaturation step at 94°C for 30 s, an annealing step at the requested temperature for 30 s, and an extension step at 72°C for 30 s. The last cycle was followed by 5-min incubation step at 72°C. Annealing temperature was determined empirically after an initial estimate based on primer length and composition. Loss of heterozygosity was analysed by polyacrylamide gel electrophoresis (PAGE) analysis. Amplified DNA was mixed with an equal volume of formamide loading dye (95% formamide, 20 mM EDTA, 0.05% bromophenol blue and 0.05% xylen cyanol), and loaded onto a gel consisting of 10–12% acrylamide (19 : 1 acrylamide : bisacrylamide), 0.089 M Tris (pH 8,3), 0.089 M borate and 0.002 M EDTA.

Samples were electrophoresed at 150 volts for 2–4 h, and the results were visualised by silver staining, using the Silver Stain kit (Bio-Rad Lab., Hercules, CA, USA). Loss of heterozygosity were visualised by using an optical densitometry scanner. For informative cases, the criteria for LOH were complete or near complete loss of one or both alleles of the DNA band corresponding to the microsatellite sequence. In particular, LOH was defined according to the following formula: LOH index=(*T*_2_/*T*_1_)/(*G*_2_/*G*_1_), where *T* was the suspicious or malignant sample, *G* was the matching goiter sample, 1 and 2 were the intensities of smaller and larger alleles, respectively. Loss of heterozygosity was considered positive in presence of values of LOH index <0.6 or >1.7. Reproducibility of each LOH was confirmed by at least two independent experiments. Constitutional homozigosity was considered as noninformative. Experiments in which allelic imbalances were detected were repeated twice. Results were reproducible in all the experiments carried out.

This study has been carried out according to the ethical guidelines of the Declaration of Helsinki. Specific authorisation was also obtained by each Institutional Scientific Board.

## RESULTS

### Presence of transformed thyrocytes and deregulated cell growth in a subset of HT

In order to investigate the presence of potentially transformed thyrocytes in HT, an extensive galectin-3 expression analysis has been done. The results of this study are shown in Table 1

**Table 1:** Galectin-3 expression in Hashimoto's thyroiditis (HTs) as evaluated immunohistochemically by using a biotin-free detection system

**Case no.**	**Galectin-3 expression**
*HTs with classic morphologic features of chronic autoimmune thyroiditis*	
1	+ Focal area with oncocytic cells
2	Neg.
3	Neg.
4	Neg.
5	Neg.
6	± Focal area with oncocytic cells
7	± Focal area with oncocytic cells
8	Neg.
9	Neg.
10	Neg.
11	Neg.
12	Neg.
13	Neg.
14	Neg.
15	Neg.
16	Neg.
17	Neg.
18	Neg.
19	Neg.
20	+ Oncocytic cells associated to lymphatic nodule
21	Neg.
22	+ Focal area with oncocytic cells
23	Neg.
24	Neg.
25	+ Scattered follicles
26	+ Scattered follicles, some with oncocytic features
27	Neg.
28	Neg.
29	Neg.
30	Neg.
31	Neg.
32	Neg.
33	Neg.
34	Neg.
35	+ Scattered follicles
36	+ Solid cell nests
37	+ Scattered follicles
38	Neg.
39	Neg.
40	Neg.
41	Neg.
42	Neg.
43	Neg.
44	Neg.
45	Neg.
46	Neg.
47	Neg.
48	Neg.
49	± Weak and focal staining of some oncocytic follicles
50	Neg.
51	Neg.
52	Neg.
53	Neg.
54	Neg.
55	Neg.
56	Neg.
57	Neg.
58	Neg.
59	Neg.
60	Neg.
61	Neg.
62	Neg
63	Neg.
64	+ Oncocytic cells associated to lymphatic nodule
65	Neg.
66	± Focal area with follicular structures and tall cells
67	Neg.
68	± Focal area with follicular structures and tall cells
69	Neg.
70	Neg.
71	Neg.
72	Neg.
73	Neg.
74	Neg.
75	Neg.
76	Neg.
77	Neg.
78	Neg.
79	Neg.
80	Neg.
81	+ Scattered oncocytic follicles
82	+ Scattered oncocytic follicles
83	+ Oncocytic follicles associated to lymphatic nodule
84	Neg.
85	Neg.
86	Neg.
87	Neg.
	
*HTs associated to hyperplastic/adenomatous lesions*	
88	± Weak staining of scattered oncocytic follicles
90	± Weak staining of oncocytic adenoma
91	Neg.
92	Neg.
93	+ Oncocytic adenoma, solid cell nests and atypical oncocytic follicles associated to lymphatic nodules^a^
94	Neg.
95	Neg.
96	Neg.
97	Neg.
	
*HTs with focal undefined/suspicious thyroid lesions*	
98	+ Follicles with clear nuclei and isolated nuclear groves
100	Neg. (atypical follicles)
101	+ Focal area with atypical follicles fibrosis and sclerosis
102	+ Scattered suspicious follicles
103	Neg. (follicles with clear nuclei)
104	+ Focal area with atypical follicles
105	Neg. (atypical follicles)
106	+ Solid cell nests and follicles associated with lymphatic nodule
107	Neg. (follicles with clear nuclei)
108	Neg. (follicles with clear nuclei)
109	Neg. (suspicious oncocytic lesion)
110	+ Suspicious follicles with tall cells associated to lymphatic nodules e scattered oncocytic follicles
111	+ Microscopic area with atypical follicles, fibrosis and sclerosis
112	+ Solid cell nests
113	Neg. (suspicious oncocytic follicles)
	
*HTs associated to thyroid malignancies*	
114	+ Microcarcinoma PTC
116	+ Microcarcinoma PTC-follicular variant
117	+ Microcarcinoma PTC
118	+ Microcarcinoma PTC-follicular variant
119	+ Microcarcinoma PTC
120	+ Microcarcinoma PTC
121	+ Microcarcinoma PTC
122	+ Microcarcinoma PTC and some scattered follicles
123	+ Microcarcinoma PTC
124	+ Microcarcinoma PTC
125	+ Microcarcinoma oncocytic type
126	+ Microcarcinoma PTC
127	+ Microcarcinoma PTC-follicular variant and atypical follicles in sclerosis
128	+ Atypical follicles with clear nuclei and oncocytic follicles associated to lymphatic nodules
129	+ Microcarcinoma PTC
130	+ Microcarcinoma PTC
131	+ Microcarcinoma PTC
132	+ Microcarcinoma PTC
133	+ Microcarcinoma PTC

a Atypical follicles are defined as thyroid follicles showing nuclear changes (clear nuclei with or without groves), which are generally observed in papillary thyroid carcinomas (PTC).

.

As expected, galectin-3 was scantily detected in the cluster of the so called ‘classic HTs’. In these lesions, a focal expression of galectin-3 was observed in 19% of the instances but it was restricted to isolated and apparently normal thyroid follicles, most of which showing oncocytic changes. Interestingly, some of these follicles were intimately associated to activated lymphoid nodules in the context of thyroid parenchyma, suggesting the possibility that such oncocytic cells may trigger a specific immune response.

In the group of HTs associated to benign proliferating lesions, galectin-3 was expressed in two oncocytic adenomas. In isolated instances, this marker was variably detected on scattered oncocytic follicles also. Hyperplastic thyroid parenchyma and residual normal parenchyma did not show any galectin-3 immunoreactivity.

**Figure 1:**
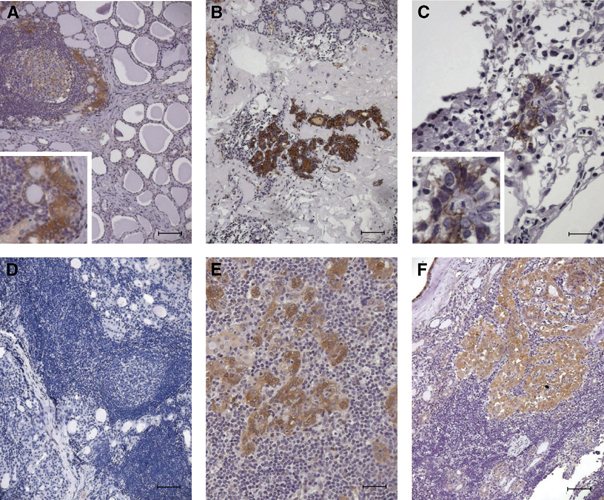
Galectin-3 expression in a subset of HTs showing focal suspicious follicular structures. Galectin-3-positive atypical thyrocytes, organized in follicles, are intimately associated to a lymphoid nodule (**A**) or localised within sclerotic areas (**B**). A single galectin-3-positive atypical thyroid follicle with tall cells. A mitosis is clearly visible in the center of the follicle (inset) (**C**). A classic type of HT was used as negative control (**D**). (**E**–**F**) Atypical galectin-3-positive follicular structures, suggestive of microcarcinomas (scale bar **A**: 43 *μ*m; **B**, **D**, **F**: 55 *μ*m; **C**, **E**: 27.5 *μ*m).

Surprisingly, in the cluster of HTs in which focal undefined/suspicious areas were morphologically detected, galectin-3 immunostaining was restricted to such suspicious epithelial structures. More then 56% of these lesions were highlighted by mAb to galectin-3, whereas the residual thyroid parenchyma was invariably galectin-3 negative (Table 1 and Figure 1A–D

).

As expected, all of the thyroid microcarcinomas discovered in the context of HT showed a homogeneous galectin-3 expression (Table 1 and Figure 1E–F).

Occult PTC represented the majority of these malignancies, with size ranging from 1–4 mm. In few instances, aspects of follicular differentiation were visible. Finally, none of the five unspecific chronic thyroiditis showed expression of galectin-3 (data not shown).

To better define the significance of the galectin-3-positive focal suspicious/atypical lesions detected in HTs, a comparative expression analysis has been crried out, considering different molecules that are known to be involved in thyroid cancer and cell cycle derangement. Results of this analysis are shown in Table 2

**Table 2:** Comparative expression of galectin-3, c-met protein, HBME-1 and cyclin D1 in a subset of HTs harbouring undefined microscopic lesions

**Case no**	**GAL-3**	**c-met**	**HBME-1**	**Cyclin-D1 (nuclear staining)**
72 (neg. control)	Neg.	+ Oncocytic follicles	Neg.	Neg.
73 (neg. control)	Neg.	+ Oncocytic follicles	Neg.	Neg.
74 (neg. control)	Neg.	+ Oncocytic follicles	Neg.	Neg.
26^a^	+ Oncocytic follicles	+ Oncocytic follicles	+ Oncocytic follicles	+ Oncocytic follicles
35		Neg.		Neg.
	+ Apparently normal follicles	+ Some oncocytic follicles	+ Apparently normal follicles	
68		Neg.		Neg.
	± Suspected follicles with tall cells	+ Some oncocytic follicles	± Suspected follicles with tall cells	
83	+ Suspected minimal oncocytic lesion	+ Suspected minimal oncocytic lesion and other oncocytic follicles	+ Suspected minimal oncocytic lesion	Neg.
93^a^	+ Atypical area and some oncocytic follicles	+ Atypical area and some oncocytic follicles	+ Atypical area	+ Atypical area
98	+ Atypical follicles	± Atypical follicles and some oncocytic follicles	+ Atypical follicles	Neg.
101	+ Atypical follicles in sclerosis	+ Atypical follicles in sclerosis and some oncocytic follicles	+ Atypical follicles in sclerosis	Neg.
102^a^	+ Atypical follicles	+ Atypical follicles and some oncocytic follicles	+ Atypical follicles	+ Atypical follicles
104	+ Suspected atypical follicles	Neg.	+ Suspected atypical follicles	Neg.
106^a^	+ Atypical follicles	+ Atypical follicles and some oncocytic follicles	+ Atypical follicles	+ Atypical follicles
110		Neg.		
	+ Suspected atypical follicles	+ Some oncocytic follicles	+ Suspected atypical follicles	NE^b^
111^a^	+ Atypical follicles and some oncocytic follicles	± Atypical follicles and some oncocytic follicles	+ Atypical follicles	+ Atypical follicles
PTC as positive control	+ PTC	+ PTC and some peritumoral follicles	+ PTC	+ PTC

a These lesions showing coexpression of markers associated to transformed thyroid cells and deregulated cell cycle control may be considered as precursors of thyroid cancer.

b NE=not evaluated.

. Cases labelled 72–74, representative of classic autoimmune HTs, were used as negative controls. Interestingly, the expression of galectin-3 in the aforementioned suspicious/atypical microscopic areas strongly correlated with the expression of HBME-1, a thyroid cancer-associated antigen.

**Figure 2:**
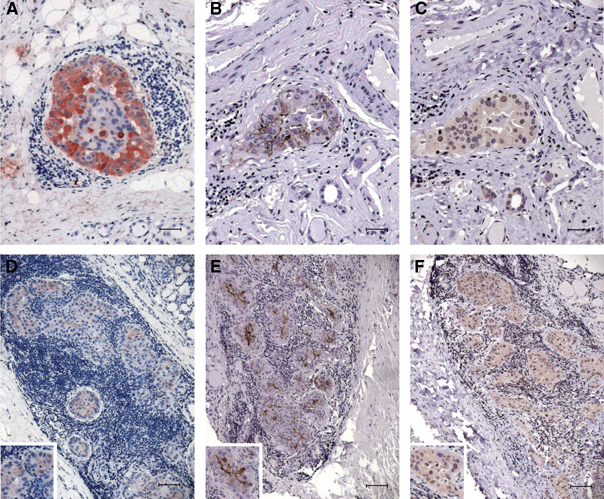
Comparative expression of galectin-3, HBME-1 and cyclin D1 in selected focal suspicious lesions detected in HTs. (**A**–**C**) Expression of galectin-3, HBME-1 and cyclin D1 in a single atypical follicle detected in HT. Galectin-3 staining is restricted to the cytoplasm of follicular cells (**A**), whereas HBME-1 is expressed on the plasma membrane with incomplete latero-lateral and apical staining pattern (**B**). On the other hand, specific cyclin D1 nuclear staining is visible in about 50% of the nuclei (**C**). (**D**–**F**) In this case, the suspicious lesion is represented by atypical follicles surrounded by intense lymphocyte infiltration. Galectin-3 is variably expressed in this lesion with a cytoplasm pattern of staining (**D**). Coexpression of HBME-1 (**E**) and cyclin D1 (**F**) is also visible (scale bar **A**–**C**: 25 *μ*m; **D**: 43 *μ*m; **E**–**F**: 55 *μ*m).

Moreover in five out of 12 cases (42%), a sharp nuclear staining for cyclin D1 was also detected, demonstrating the presence in these instances of both thyrocytes transformation and deregulated cell cycle control (Figure 2A–F

).

Expression of c-met protein, which is generally observed in well-differentiated PTC, partially correlated with the expression of galectin-3 and HBME-1 (66% of the cases). Expression of c-met protein was also observed in some follicles showing oncocytic features without any morphological evidence of malignancy. These follicular structures were found associated to infiltrating lymphocytes or in the context of activated lymphocyte nodules.

The significance of this finding, observed in some instances in galectin-3 immunostaining also, is under investigation.

As expected, almost all the thyroid papillary carcinomas tested as controls showed coexpression of galectin-3, HBME-1, c-met protein and cyclin D1 (data not shown). A PTC case was included in Table 2 as positive control.

All together these findings suggest that specific subsets of HTs may harbour potential thyroid cancer's precursors.

### Loss of heterozygosity at specific chromosomal loci in Hashimoto's thyroiditis

**Figure 3:**
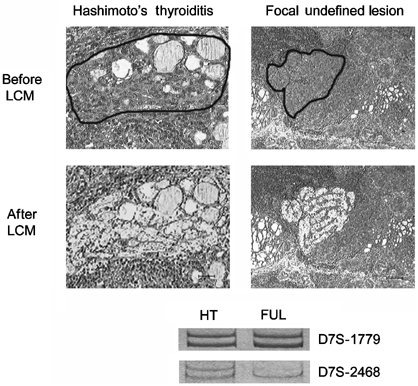
Laser capture microdissection (LCM) and of LOH for microsatellite D7S-2468 in a focal galectin-3-positive undefined lesion (FUL) detected in HT. The upper panel shows a classic case of HT and a focal undefined lesion detected in HT before and after laser microdissection. Note that galectin-3 expression was restricted to the FUL (dark staining). The black line outlines the microdissected areas. The lower panel shows an LOH for microsatellite D7S-2468 in the galectin-3-positive lesion. (scale bar HT: 43 *μ*m; FUL: 55 *μ*m).

Specific allelic loss for microsatellite D7S-2468 was demonstrated in three out of three focal undefined lesions (FULs) associated to HTs and in four out of six foci of micro-PTCs discovered in such diseases, by using laser capture microdissection followed by DNA extraction and PCR. Two cases of classical HTs were used as negative controls (Figure 3

and data not shown). All the HTs cases considered in this analysis were previously assessed for expression of galectin-3.

Polymerase chain reactions were carried out in the presence of specific primers flanking the sequence of two microsatellite markers D7S-1779 and D7S-2468 located at the chromosomal region 7q32–34 (see Material and methods section for detail). Loss of heterozygosity for the specific microsatellite region was restricted to galectin-3-positive thyrocytes microdissected from the aforementioned suspicious or malignant areas, while thyroid cells isolated from the surrounding galectin-3-negative parenchyma, as well as from the thyroid follicles of classic HTs, retained heterozygosity (Figure 3).

These findings indicated that loss of genetic material, and in particular LOH at a critical region of the long arm of chromosome 7 (7q32–34) was associated to both morphologic and phenotypic alterations of thyrocytes.

All together these morphological, phenotypical and molecular findings suggest that at least some of the FULs observed in HTs may likely represent thyroid cancer's precursors.

## DISCUSSION

### The pathogenetic variability of HT

Many immunological and genetic studies have been carried out in order to elucidate the pathophysiology of HT. Intriguing experimental evidences from two independent groups suggested that apoptosis may be responsible for the thyroid cells destruction observed in HT (Giordano *et al*, 1997; Hammond *et al*, 1997; Stassi and De Maria, 2002). The proposed mechanism involves Fas, a member of the tumour necrosis factor receptor family. Cross-linking of Fas with its specific ligand (FasL) is sufficient to induce apoptosis in the Fas-bearing cells. Although Fas is mainly expressed on activated T lymphocytes and natural killer cells, which are the effectors of the immunoresponse against viruses and tumours, follicular thyroid cells in HT have been also found to express Fas on their cell surface. Fas expression is induced by IL-1*β*, a cytokine produced by activated lymphocytes. Fas-positive target thyrocytes are killed when exposed to FasL-positive neighbouring thyroid cells. Although Fas–FasL-mediated apoptosis in thyrocytes is probably not so simple as originally conceived, this mechanism may likely explain the determinism of thyroid atrophy and hypothyroidism observed in a subset of HTs (Dayan *et al*, 1997; Mirakian *et al*, 1998). However, from the immunological point of view, other mechanisms could be involved in the pathogenesis of HT. Autoimmune diseases represent an abnormal recognition of self-antigens by activated CD4+ (helper) T lymphocytes. In this context, HT may be triggered by at least three different mechanisms. The first, referred as ‘molecular mimicry’, is supported by the serological evidence of bacterial or viral infection in patients with HT.

A common epitope of an infectious antigen, similar to a thyroid protein, could results in activation of specific T lymphocytes (Davies *et al*, 1991; Zhao *et al*, 1998).

An alternative mechanism implies the loss of self-tolerance that may result from defective deletion or anergy of specific T lymphocytes, targeted against self-antigens. Similar effects may be generated by loss of suppression of self-reactive T cells by regulatory cells secreting immunosuppressive cytokines (Walker and Abbas, 2002). A third mechanism of cell injury is based on the assumption that thyroid cells may present their own intracellular proteins to CD4+ T lymphocytes. This view is supported by the finding that thyroid cells during thyroiditis may express the major histocompatibility complex (MHC) class II proteins, a critical structure for antigen presentation to CD4+ T lymphocytes. Furthermore, interferon-*γ* released from activated T lymphocytes can induce the expression of MHC class II molecules on thyroid cells, leading T-cell restimulation and perpetuation of the autoimmune process (Huang and Kukes, 1999). Once activated, self-reactive CD4+ T lymphocytes can stimulate autoreactive B cells to secrete thyroid autoantibodies, among which those directed to thyroglobulin (TG), thyroperoxidase (TPO) and thyrotropin receptor (TSHr) are better known. Together with the possibility that thyroid cells can present self-antigens to CD4+ T lymphocytes, it should be considered that mutational events inducing thyrocytes transformation may potentially drive the expression of aberrant proteins on the cell surface (Powell *et al*, 2003). This mechanism represents *per se* a condition that is necessary and sufficient for triggering an active immunological response to still undefined neoexpressed antigens. The Chernobyl's disaster strongly supports this possibility. Pacini *et al* (1998) reported that post-Chernobyl Belarus thyroid carcinomas were frequently associated with ‘thyroid autoimmunity’. Thyroid lymphocyte infiltration and circulating anti-TPO antibodies, in fact, were consistently detected in Belarus patients with respect to the controls (Pacini *et al*, 1998). Moreover, oncocytic changes of thyroid cells have been reported among the late chronic thyroid alterations observed after Chernobyl disaster, with a frequency ranging from 17 to 42% (Nikiforov and Gnepp, 1999). These clinical-pathological pictures of chronic autoimmune thyroiditis are very similar to those observed in HT.

It is intriguing to speculate that in these thyroid conditions, a specific immune response to still undefined aberrant proteins may be induced by radiation damages. This interpretation is fully in line with the observation of both autoimmune thyroiditis and increased risk of thyroid cancer in survivors of the atomic bomb blast, as well as in subjects exposed to radiation fallout in the Marshall Islands (Nagataki *et al*, 1994). These human tragedies provide *per se* an unequivocal demonstration that a subset of chronic autoimmune thyroiditis (with histological features of HTs) may harbour transformed thyrocytes and can be triggered by a qualitatively different immune response. Although these facts can explain at least in part the origin of HTs, we should consider that the aforementioned pathogenetic mechanisms are sustainable also. For these reasons, we believe that HTs should be considered more properly as a spectrum of different thyroid conditions rather than as a single and well-defined clinical–pathological entity.

### Hashimoto's thyroiditis may harbour potential precursor lesions of thyroid cancer

The morphological and phenotypic analysis reported here provides the evidence that a subset of HTs may harbour potential thyroid cancer's precursors. This concept is substantiated by the finding that a gradient of expression of galectin-3, a well-know molecule associated to thyrocytes transformation, has been observed in different clusters of morphologically categorised HTs. Interestingly, the most relevant expression of galectin-3 was specifically detected in those HTs harbouring focal suspicious areas, as well as in almost all microcarcinomas discovered in HT (Bartolazzi *et al*, 2001; Takenaka *et al*, 2003; Yang and Liu, 2003). A panel of galectin-3-positive suspicious lesions has been also assessed for the comparative expression of HBME-1, c-met protein and cyclin D1 (Hall and Peters, 1996; Miettinen and Karkkainen, 1996; Ruco *et al*, 2001).

HBME-1 identifies a monoclonal antibody directed to a biochemical unknown epitope, which is consistently expressed in thyroid cancer (Miettinen and Karkkainen, 1996). Both galectin-3 and HBME-1 are commonly used in clinical practice as diagnostic tools for improving the accuracy of conventional histomorphological methods (Miettinen and Karkkainen, 1996; Bartolazzi *et al*, 2001). On the other hand, overexpression of c-met protein has been reported as a distinguish feature of well-differentiated papillary carcinoma (Ruco *et al*, 2001).

With regard to cyclin D1, it is a well-known regulator of cell cycle and its overexpression plays an important role in tumour progression (Hall and Peters, 1996; Wang *et al*, 2000). Cyclin D1 forms a multimeric complex with cyclin-dependent kinase, which facilitate transition through the restriction point of cell cycle by inactivation of the retinoblastoma tumour-suppressor protein (pRb). Constitutive overexpression of cyclin D1, caused by chromosomal abnormalities, gene amplification or other post-translational mechanisms has been reported in different types of tumours including thyroid cancer (Hall and Peters, 1996; Wang *et al*, 2000). For these reasons, the concurrent expression of galectin-3, HBME-1, c-met protein and cyclin D1 observed in some suspicious lesions in the context of HTs strongly suggests the presence of transformed thyrocytes.

Genetic and exogenous factors have also been involved in the determinism of HT. It is noteworthy that proliferative lesions associated with HT, but not the perilesional tissue involved by the inflammatory infiltration, may be karyotypically abnormal (Vanni *et al*, 2000). This means that cytogenetic changes are likely due to the derangement of the cell cycle regulatory machinery rather than to the inflammatory parenchymal alterations. Although the detection of molecular alterations in HT, such as the presence of the papillary carcinoma-related fusion gene RET/PTC, is still a matter of discussion, the loss of genetic material at specific chromosomal loci has been demonstrated by Hunt and co-workers (Wirtschafter *et al*, 1997; Hunt *et al*, 2002; Nikiforova *et al*, 2002). We show here that LOH for a microsatellite located in the chromosomal region 7q32–34, previously reported in PTC, also occurs in some galectin-3-positive focal undefined lesions detected in HTs. Although further investigations are necessary to better clarify the biological significance of these findings, the phenotypical and molecular alterations reported here strongly suggest the presence of thyroid cancer precursors in a subset of HTs. If it is true that the clinical impact of early detection of thyroid cancer precursors and/or occult thyroid carcinomas in HTs must be demonstrated and will be matter of discussions, it is unquestionable that the concept of ‘Hashimoto's thyroiditis’ as a single clinical–pathologic entity should be revisited. The subclassification of HTs that we propose provides the basis for further clinical, pathological and molecular investigations aimed to better understand the clinical outcome and to improve the management of these heterogeneous thyroid conditions.

**Figure 4:**
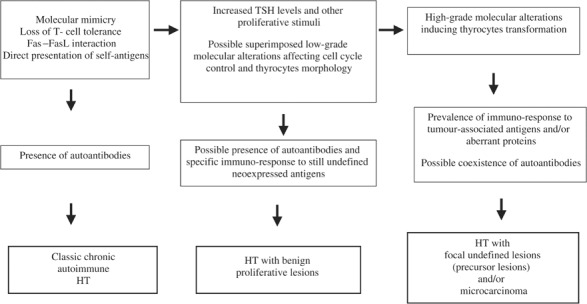
Preliminary pathogenetic hypothesis for the different clusters of HTs identified in this study.

A preliminary attempt to establish a pathophysiological model, which can explain, at least in part, the spectrum of the thyroid conditions that we observed is shown in Figure 4

. Further experimental and clinical investigations will certainly contribute to better understand when HT will progress towards a complete atrophy of thyroid parenchyma and hypothyroidism, when tumours will arise and when a chronic autoimmune thyroiditis will be indolent over the years.

## References

[bib1] Bartolazzi A, Gasbarri A, Papotti M, Bussolati G, Lucante T, Khan A, Inohara H, Marandino F, Orlandi F, Nardi F, Vecchione A, Tecce R, Larsson O, and Thyroid Cancer Study Group (2001) Application of an immunodiagnostic method for improving the preoperative diagnosis of nodular thyroid lesions. Lancet 357: 1644–1650 11425367 10.1016/s0140-6736(00)04817-0

[bib2] Davies TF, Martin A, Concepcion ES, Graves P, Cohen L, Ben-Nun A (1991) Evidence of limited variability of antigen receptors on intra-thyroidal T cells in autoimmune thyroid disease. N Engl J Med 325: 238–244 1829139 10.1056/NEJM199107253250404

[bib3] Dayan CM, Daniels GH (1996) Chronic autoimmune thyroiditis. N Engl J Med 335: 99–107 8649497 10.1056/NEJM199607113350206

[bib4] Dayan CM, Elsegood KA, Maile R (1997) FasL expression on epithelial cells: the Bottazzo–Feldman hypothesis revisited. Immunol Today 18: 203–207 9153948 10.1016/s0167-5699(97)81661-1

[bib5] Giordano C, Stassi G, De Maria R, Todaro M, Richiusa P, Papoff G, Ruberti G, Bagnasco M, Testi R, Galluzo A (1997) Potential involvement of Fas and its ligand in the pathogenesis of Hashimoto's thyroiditis. Science 275: 960–963 9020075 10.1126/science.275.5302.960

[bib6] Hall M, Peters G (1996) Genetic alterations of cyclins, cyclin-dependent kinases, and cdk inhibitors in human cancer. Adv Cancer Res 68: 67–108 8712071 10.1016/s0065-230x(08)60352-8

[bib7] Hammond LJ, Lowdell MW, Goode AW, Bottazzo GF, Mirakian R (1997) Analysis of apoptosis in relation to tissue destruction associated with autoimmune thyroiditis. J Pathol 182: 138–144 9274522 10.1002/(SICI)1096-9896(199706)182:2<138::AID-PATH810>3.0.CO;2-F

[bib8] Hashimoto H (1912) Zur kenntniss der lymphomatosen veranderung der schilddruse (struma lymphomatosa). Arch Klin Chir 97: 219–248

[bib9] Hayashi Y, Tamai H, Fukata S, Hirota Y, Katayama S, Kuma K, Kumagai LF, Nagataki S (1985) A long term clinical, immunological, and histological follow-up study of patients with goitrous chronic lymphocytic thyroiditis. J Clin Endocrinol Metab 61: 1172–1178 3840493 10.1210/jcem-61-6-1172

[bib10] Huang W, Kukes GD (1999) Hashimoto's thyroiditis: an organ-specific autoimmune disease. Pathogenesis and recent developments. Lab Invest 79: 1175–1180 10532582

[bib11] Huang Y, Prasad M, Lemon WJ, Hampel H, Wright FA, Kornacker K, LiVolsi V, Frankel W, Kloos RT, Eng C, Pellegata NS, de la Chapelle A (2001) Gene expression in papillary thyroid carcinoma reveals highly consistent profiles. Proc Natl Acad Sci USA 98: 15044–15049 11752453 10.1073/pnas.251547398 PMC64980

[bib12] Hunt JL, Baloch ZW, Barnes L, Swalsky PA, Trusky CL, Sesatomi E, Finkelstein S, LiVolsi VA (2002) Loss of heterozygosity mutations of tumor suppressor genes in cytologically atypical areas in chronic lymphocytic thyroiditis. Endocr Pathol 13: 321–330 12665650 10.1385/ep:13:4:321

[bib13] Miettinen M, Karkkainen P (1996) Differential reactivity of HBME-1 and CD15 antibodies in benign and malignant thyroid tumours. Preferential reactivity with malignant tumours. Virchows Arch 429: 213–219 8972756 10.1007/BF00198336

[bib14] Mirakian R, Hammond LJ, Bottazzo GF (1998) Pathogenesis of thyroid autoimmunity: the Bottazzo–Feldmann hypothesis. Immunol Today 9: 97–98 9509765

[bib15] Nagataki S, Shibata Y, Inoue S, Yokoyama N, Izumi M, Shimaoka K (1994) Thyroid disease among atomic bomb survivors in Nagasaki. JAMA 272: 364–370 8028167

[bib16] Nikiforova MN, Caudill CM, Biddinger P, Nikiforov YE (2002) Prevalence of RET/PTC rearrangements in Hashimoto's thyroiditis and papillary thyroid carcinomas. Int J Surg Pathol 10: 15–22 11927965 10.1177/106689690201000104

[bib17] Nikiforov YE, Gnepp DR (1999) Pathomorphology of thyroid gland lesions associated with radiation exposure: the Chernobyl experience and review of the literature. Adv Anat Pathol 6: 78–91 10331070 10.1097/00125480-199903000-00002

[bib18] Pacini F, Vorontsova T, Molinaro E, Kuchinskaya E, Agate L, Shavrova E, Astachova L, Chiovato L, Pinchera A (1998) Prevalence of thyroid autoantibodies in children and adolescents from Belarus exposed to the Chernobyl radioactive fallout. Lancet 352: 1–4 9737280 10.1016/s0140-6736(97)11397-6

[bib19] Powell Jr DJ, Eisenlohr LC, Rothstein JL (2003) A thyroid tumor-specific antigen formed by the fusion of two self proteins. J Immunol 170: 861–869 12517951 10.4049/jimmunol.170.2.861

[bib20] Roitt IM, Doniach D, Campbell PN, Hudson RV (1956) Auto-antibodies in Hashimoto's disease (lymphadenoid goitre). Lancet 2: 820–821 10.1016/s0140-6736(56)92249-8 13368530

[bib21] Roque L, Nunes VM, Ribeiro C, Martins C, Soares J (2001) Karyotypic characterization of papillary thyroid carcinomas. Cancer 92: 2529–2538 11745186 10.1002/1097-0142(20011115)92:10<2529::aid-cncr1604>3.0.co;2-m

[bib22] Ruco PL, Stoppacciaro A, Ballarini F, Prat M, Scarpino S (2001) Met protein and hepatocyte growth factor (HGF) in papillary carcinoma of the thyroid: evidence for a pathogenetic role in tumourigenesis. J Pathol 194: 4–8 11329134 10.1002/path.847

[bib23] Stassi G, De Maria R (2002) Autoimmune thyroid disease: new models of cell death in autoimmunity. Nat Rev Immunol 2: 195–204 11913070 10.1038/nri750

[bib24] Takenaka Y, Inohara H, Yoshii T, Oshima K, Nakahara S, Akahani S, Honjo Y, Yamamoto Y, Raz A, Kubo T (2003) Malignant transformation of thyroid follicular cells by galectin-3. Cancer Lett 195: 111–119 12767519 10.1016/s0304-3835(03)00056-9

[bib25] Vanni R, Marras-Virdis S, Gerosa C, Lai ML, Tallini G (2000) Cytogenetics of thyroid nodules in Hashimoto thyroiditis. Cancer Genet Cytogenet 120: 87–88 10939845 10.1016/s0165-4608(99)00244-7

[bib26] Vickery AL, Hamblin Jr E (1961) Struma lymphomatosa (Hashimoto's thyroiditis): observations on repeated biopsies in sixteen patients. N Engl J Med 264: 226–229 13781168 10.1056/NEJM196102022640505

[bib27] Walker LS, Abbas AK (2002) The enemy within: keeping self-reactive T cells at bay in the periphery. Nat Rev Immunol 2: 11–19 11908514 10.1038/nri701

[bib28] Wang S, Lloyd RV, Hutzler MJ, Safran MS, Patwardhan NA, Khan A (2000) The role of cell cycle regulatory protein, cyclin D1, in the progression of thyroid cancer. Mod Pathol 13: 882–887 10955455 10.1038/modpathol.3880157

[bib29] Wirtschafter A, Schmidt R, Rosen D, Kundu N, Santoro M, Fusco A, Multhaupt H, Atkins JP, Rosen MR, Keane WM, Rothstein JL (1997) Expression of the RET/PTC fusion gene as a marker for papillary carcinoma in Hashimoto's thyroiditis. Laryngoscope 107: 95–100 9001272 10.1097/00005537-199701000-00019

[bib30] Wreesmann VB, Ghossein RA, Patel SG, Harris CP, Schnaser EA, Shaha AR, Tuttle RM, Shah JP, Rao PH, Singh B (2002) Genome-wide appraisal of thyroid cancer progression. Am J Pathol 161: 1549–1556 12414503 10.1016/S0002-9440(10)64433-1 PMC1850764

[bib31] Yang RY, Liu FT (2003) Galectins in cell growth and apoptosis. Cell Mol Life Sci 60: 267–276 12678492 10.1007/s000180300022 PMC11138630

[bib32] Zhang JS, Nelson M, Mc Iver B, Hay ID, Goellner JR, Grant CS, Eberhardt NL, Smith DI (1998) Differential loss of heterozygosity at 7q31.2 in follicular and papillary thyroid tumors. Oncogene 17: 789–793 9715281 10.1038/sj.onc.1201996

[bib33] Zhao ZS, Granucci F, Yeh L, Schaffer PA, Cantor H (1998) Molecular mimicry by herpes simplex virus-type 1: autoimmune disease after viral infection. Science 279: 1344–1346 9478893 10.1126/science.279.5355.1344

